# Computational and Serologic Analysis of Novel and Known Viruses in Species Human Adenovirus D in Which Serology and Genomics Do Not Correlate

**DOI:** 10.1371/journal.pone.0033212

**Published:** 2012-03-13

**Authors:** Elizabeth B. Liu, Debra A. Wadford, Jason Seto, Maria Vu, Nolan Ryan Hudson, Lisa Thrasher, Sarah Torres, David W. Dyer, James Chodosh, Donald Seto, Morris S. Jones

**Affiliations:** 1 School of Systems Biology, George Mason University, Manassas, Virginia, United States of America; 2 Viral and Rickettsial Disease Laboratory, California Department of Public Health, Richmond, California, United States of America; 3 Clinical Investigation Facility, David Grant USAF Medical Center, Travis AFB, Fairfield, California, United States of America; 4 Department of Microbiology and Immunology, University of Oklahoma Health Sciences Center, Oklahoma City, Oklahoma, United States of America; 5 Howe Laboratory, Massachusetts Eye and Ear Infirmary, Department of Ophthalmology, Harvard Medical School, Boston, Massachusetts, United States of America; Mayo Clinic, United States of America

## Abstract

In November of 2007 a human adenovirus (HAdV) was isolated from a bronchoalveolar lavage (BAL) sample recovered from a biopsy of an AIDS patient who presented with fever, cough, tachycardia, and expiratory wheezes. To better understand the isolated virus, the genome was sequenced and analyzed using bioinformatic and phylogenomic analysis. The results suggest that this novel virus, which is provisionally named HAdV-D59, may have been created from multiple recombination events. Specifically, the penton, hexon, and fiber genes have high nucleotide identity to HAdV-D19C, HAdV-D25, and HAdV-D56, respectively. Serological results demonstrated that HAdV-D59 has a neutralization profile that is similar yet not identical to that of HAdV-D25. Furthermore, we observed a two-fold difference between the ability of HAdV-D15 and HAdV-D25 to be neutralized by reciprocal antiserum indicating that the two hexon proteins may be more similar in epitopic conformation than previously assumed. In contrast, hexon loops 1 and 2 of HAdV-D15 and HAdV-D25 share 79.13 and 92.56 percent nucleotide identity, respectively. These data suggest that serology and genomics do not always correlate.

## Introduction

The first human adenoviruses (HAdVs) were isolated in 1953 from a military basic trainee and the adenoid tissue of a pediatric patient as respiratory pathogens [1]. Presently there are greater than 60 types that have been isolated and characterized either with serological methods or, more recently, genomic methods [2], [3], [4], [5], [6]. HAdVs are classified into the Mastadenovirus genus and are further parsed into seven species (A–G) [3], [4], [5], [6], [7], [8]. Originally, HAdV types were identified, characterized and classified based on serum neutralization and hemagglutination inhibition assays among other biological attributes [9]; however recently, bioinformatics and genomic analysis of the whole genome have replaced serology-based methods for typing novel HAdVs [3], [4], [5], [6], [8], [10]. At the nucleotide level, the members of each adenovirus species are highly similar to each other, and do not commonly recombine with members of other species. The species groupings, in part, may reflect the cell tropism of the viruses, as well as the resulting symptoms and diseases caused by the individual HAdV types. For example, species HAdV-B1 viruses are known to cause respiratory infections of the lower lung [11] whereas viruses in species HAdV-D can cause ocular disease, including epidemic keratoconjunctivitis [12], and gastrointestinal disease [3], [13].

In this report, an adenovirus isolated from a bronchoalveolar lavage (BAL) sample that was biopsied from an AIDS patient who presented with fever, cough, tachycardia and expiratory wheezes is examined using genomics and bioinformatics. Based upon the whole genome analysis and supported by limited serological data, this adenovirus belongs to species HAdV-D, and is a ‘never seen before’ novel virus, to be given the name of HAdV-D59.

## Materials and Methods

### Ethics Statement

The work reported herein was performed under United States Air Force Surgeon General-approved Clinical Investigation No. FDG20040024E, by the Institutional Review Board at the David Grant USAF Medical Center. Informed Consent was not required, because we did not use clinical samples.

### Viruses, cells, and serum neutralization assay

Adenovirus neutralization assays were run as previously described [14]. Briefly, serotyping of adenovirus isolates was performed using a standard dose of virus against specific rabbit antisera raised against reference stock adenoviruses types 1–49 from the collection maintained by the Viral and Rickettsial Disease Laboratory of the California Department of Public Health, Richmond, CA. Reference viruses were originally obtained from the reporting investigators: the Research Reference Reagents Branch, National Institutes of Health; the Respiratory Viral Disease Unit, Centers for Disease Control and Prevention; or the American Type Culture Collection. Stock virus cultures were passaged in A549 cells (American Type Culture Collection, Rockville, MD), the cells were disrupted by vortexing, and cell-free supernatant fluid was then frozen at −70°C.

Equal volumes of diluted virus and immune serum were mixed and incubated for one hour in 5% CO_2_ at 37°C. Thereafter A549 cells were added, mixed, and incubated at 37°C in 5% CO_2_ for 7 days. Each assay contained a back titration of the virus used. Living cells were distinguished from dead cells by measuring the amount of Finter's Neutral Red [15] present as indicated by absorbance at 550 nm using a microplate spectrophotometer (Bio-Tek Instruments, Winooski, VT). Virus neutralization titers were determined by equating cell death to virus growth (no virus neutralization). Neutralization was plotted as a percentage of cell control absorbance, to determine endpoint virus and serum titers. Three independent experiments were run yielding similar results.

### Nucleotide sequence accession numbers

The HAdV-D59 genome sequence and its annotation are deposited in GenBank and retrievable as accession number JF799911. In addition, the following HAdV genomes (GenBank accession numbers) were used for comparative computational analyses: HAdV-D8 (AB448767), HAdV-D9 (AJ854486), HAdV-D15 (AB562586), HAdV-D17 (AF108105), HAdV-D19C (EF121005), HAdV-D22 (FJ404771), HAdV-D25 (unpublished), HAdV-D26 (EF153474), HAdV-D28 (FJ824826), HAdV-D36 (GQ384080), HAdV-D37 (DQ900900), HAdV-D46 (AY875648), HAdV-D48 (EF153473), HAdV-D49 (DQ393829), HAdV-D53 (FJ169625), HAdV-D54 (AB333801), HAdV-D56 (HM770721), and HAdV-D58 (HQ883276).

Fiber coding sequences used for analysis were as follows: HAdV-D8 (AB448767), HAdV-D9 (AJ854486), HAdV-D10 (AB369368), HAdV-D19C (AB448774), HAdV-D19a (CS301726), HAdV-D22 (FJ619037), HAdV-D26 (EF153474), HAdV-D28 (FJ824826), HAdV-D29 (AB562587), HAdV-D36 (GQ384080), HAdV-D37 (DQ900900), HAdV-D46 (AY875648), HAdV-D48 (EF153473), HAdV-D49 (DQ393829), HAdV-D54 (AB333801), HAdV-D56 (HM770721), and HAdV-D58 (HQ883276). Fiber genes from species HAdV-D genomes were aligned using ClustalW [16]. For this analysis, the default gap opening and gap extension penalties were 15.0 and 6.66.

### Amplification and DNA sequencing of the HAdV-D59 genome

To amplify regions of HAdV-D59 using the polymerase chain reaction (PCR) protocol, conserved adenovirus sequences in species HAdV-D were used to design primers. All amplicons were then sequenced on an ABI 3130xl using a primer walking strategy. The HAdV-D59 genome was sequenced to 8-fold coverage following PCR amplification, with both strands represented.

### Bioinformatics

The HAdV-D59 genome was compared against a select number of viral genomes from the HAdV-D group based on its GC content, which is indicative of HAdV species. The selection of which genomes was based on initial overall high nucleotide identity to HAdV-D59. The data presented are final iterations of analyses that initially included all of the sequenced genomes in species HAdV-D.

### Recombination analysis

Whole genome sequences of HAdV-D59 and members of species HAdV-D were first aligned with kalign (http://www.ebi.ac.uk/Tools/msa/kalign/) for a broad perspective of the genome. SimPlot [17] was then used to construct a Bootscan analysis of the aligned sequences. The window size and step size were set to 1000 and 200 respectively.

Following this, to provide a detailed close inspection of recombination events, the penton base gene, hexon gene, E3 coding region and fiber gene from the HAdV-D genomes were aligned to their counterparts using ClustalW [16]. This was also followed by recombination analysis using SimPlot with the window size and step size set to 250 and 50, respectively.

### Percent Identity

Whole genome, penton base gene, hexon gene, E3 coding region and fiber gene nucleotide sequences of HAdV-D59, along with members of species HAdV-D, were aligned using kalign; these were then compared to each other based on percent identity values calculated with Chimera [18].

### Phylogenomic analysis of HAdV-D59

Sequence alignments for phylogenomic analysis were generated using the kalign method noted earlier. Phylogenetic trees were constructed from these aligned sequences using Molecular Genetic Analysis Software (MEGA 4.1; http://www.megasoftware.net), via neighbor-joining methods and bootstrap test of phylogeny with replicates set to 1000.

## Results

### Clinical Investigation

In November 2007, an AIDS patient was admitted to San Francisco General Hospital, presenting with fever. The patient also complained of a cough productive of yellow sputum and blood. Clinical examination revealed a body temperature of 101°F, tachycardia and expiratory wheezes. During the hospital stay, a CT scan displayed results suggestive of a cavitary lung lesion. This prompted a bronchoalveolar lavage (BAL) for a diagnostic specimen (via bronchoscopy). A virus was cultured from the BAL sample and sent to the California Department of Public Health (CDPH) for further analysis and identification. No other pathogens were isolated from this patient.

The virus was propagated at the Viral and Rickettsial Disease Laboratory at the California Department of Public Health, and was identified as an unknown adenovirus by serum neutralization assay. Initial sequence analysis of amplicons derived from the hexon and the fiber genes revealed similarity to gene sequences from HAdV-D25 and HAdV-D56, respectively. The possibility that this virus might represent a novel, recombinant pathogen provoked whole genome analysis in order to characterize this isolate more thoroughly.

### Amplification, sequencing, and genetic characteristics of the novel adenovirus

**Figure 1:**
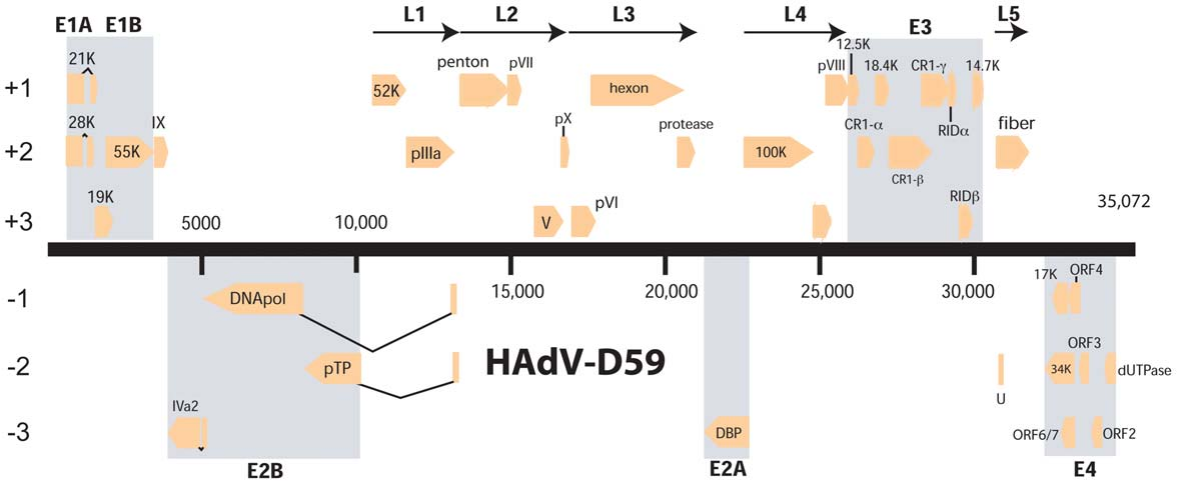
Genome organization of HAdV-D59. The HAdV-D59 genome is represented by a black horizontal line marked at 5-kbp intervals. Protein encoding regions are shown as arrows indicating transcriptional orientation above and below the genome. Spliced genes are indicated by V-shaped lines.

To elucidate the genetic characteristics of this pathogen (HAdV-D59), we sequenced and analyzed the entire genome. The genome length of HAdV-D59 is 35,072 base pairs (Figure 1), with a base composition of 22.4% A, 20.4% T, 28.5% G, 28.7% C. The GC content of 57.2% is consistent with the values found for members of species HAdV-D (mean of 57.0%). The organization of the 36 open reading frames (ORFs) that were annotated had a genome organization similar to other mastadenoviruses (Fig. 1). The inverted terminal repeat (ITR) sequences for HAdV-D59 were determined to be 151 bp in length. Within species HAdV-D, HAdV-D59 has a genome percent identity ranging from a low of 92.06% (HAdV-D8) to 96.48% (HAdV-D9).

### Genome Analysis

**Figure 2:**
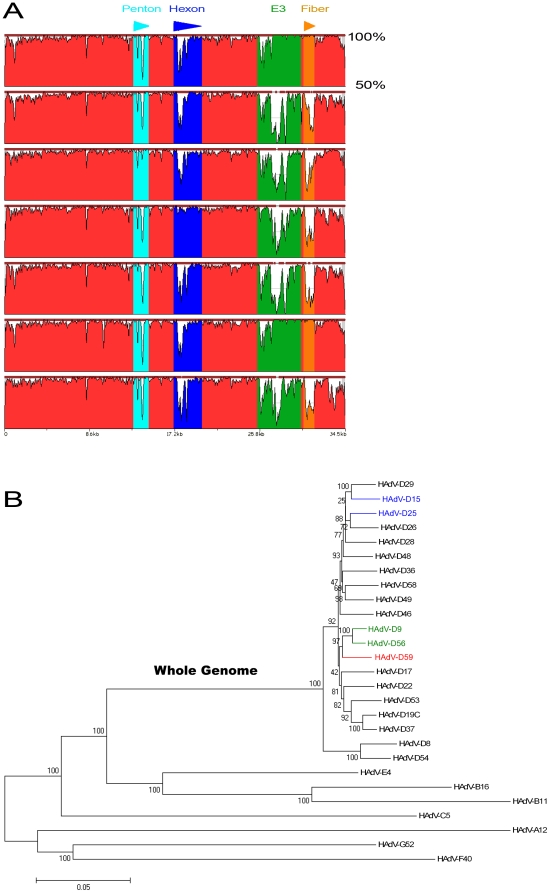
Comparative genomic analysis. (A) Pairwise nucleotide comparison of selected HAdV-D genomes to HAdV-D59 using zPicture. The arrows above the x-axis demarcate the positions of penton base, hexon, E3 region, and fiber coding sequences in the genome of HAdV-D59. The y-axis notes the percent identity. HAdV-D9, HAdV-D22, HAdV-D19C, HAdV-D25, HAdV-D28, HAdV-D36, and HAdV-D56 were used for comparison to HAdV-D59 because they share high nucleotide identity to the aforementioned virus in different sections of their genomes. (B) Whole genome phylogenetic analysis. The phylogenetic tree was constructed from aligned sequences using MEGA, via the neighbor-joining methods and a bootstrap test of phylogeny. Bootstrap values shown at the branching points indicate the percentages of 1000 replications produced the clade. A Bootstrap value of 70 and above is considered to be robust.

Since initial DNA sequencing suggested evidence of recombination, we analyzed the HAdV-D59 genome using zPicture, a dynamic blastz alignment visualization program designed for comparative analysis (http://zpicture.dcode.org/). Comparisons of the HAdV-D59 genome with the whole genome sequences of HAdV-D9, -D22, -D19C, -D25, -D28, -D36, -D56 and -D58 were performed. Consistent with other previously reported viruses in species HAdV-D [4], [19], HAdV-D59 shows heterogeneity in the penton base, hexon, E3, and fiber coding sequences (Fig. 2A). Pairwise alignment suggested that these regions had highest nucleotide identities with sequences from HAdV-D9, HAdV-D19C, HAdV-D25, HAdV-D28, and HAdV-D56 (Table 1).

**Table 1:** Percent identities of the nucleotide coding sequences of selected HAdV-D59 coding regions to homologous sequences from other viruses in species HAdV-D.

	Penton	Hexon L1	Hexon L2	E3	Fiber
HAdV-D9	92.63%	79.14%	85.56%	**96.35%**	**99.54%**
HAdV-D15	93.03%	78.10%	91.48%	80.57%	77.45%
HAdV-D19C	93.03%	75.41%	88.89%	80.52%	76.59%
HAdV-D22	**97.55%**	78.23%	81.85%	83.02%	70.75%
HAdV-D25	92.64%	**96.48%**	**95.91%**	82.82%	71.06%
HAdV-D28	92.31%	77.44%	82.22%	77.61%	69.97%
HAdV-D36	93.60%	75.55%	75.19%	78.30%	69.89%
HAdV-D56	92.44%	78.23%	91.11%	**95.98%**	**99.82%**
HAdV-D58	92.63%	74.97%	77.29%	82.88%	70.61%

Comprehensive phylogenomic analyses of whole genome HAdVs were performed. Using sequences available in GenBank as well as the unpublished sequence of HAdV-D25, the whole genome phylogenetic tree analysis resulted in a subclade that includes HAdV-D59, HAdV-D9, and HAdV-D56 with a high confidence bootstrap value of 97 (Fig. 2B).

### Penton Base Gene Analysis

**Figure 3:**
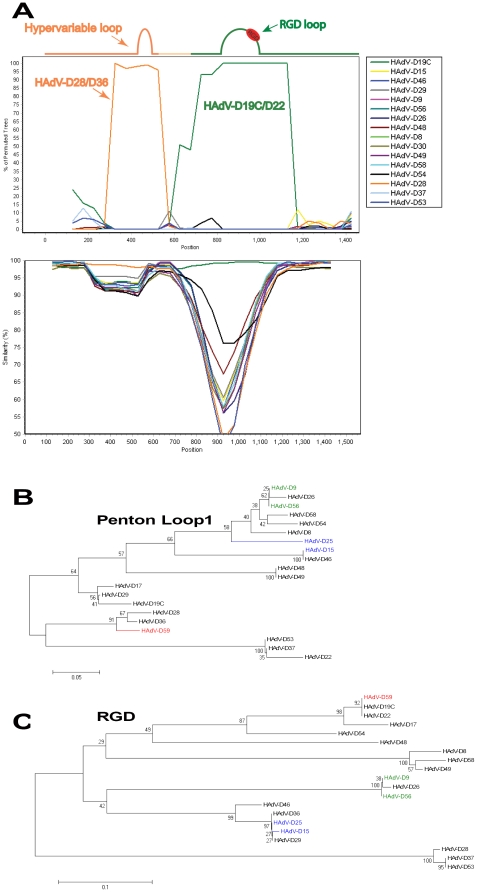
Computational analysis of the penton base gene. (A) Bootscan analysis of the HAdV-D59 penton base gene with fully sequenced penton genes in species HAdV-D using a window size of 250 bp and step size of 50 bp. (B) Phylogenetic analysis of the hypervariable loop 1 penton base gene sequences in species HAdV-D. (C) Phylogenetic analysis of the RGD motif and surrounding variable region of sequences in species HAdV-D. The phylogenetic trees were generated from aligned sequences using MEGA, via the neighbor-joining method and a bootstrap test of phylogeny.

Recently it was shown that two coding sequences for the external hypervariable loops in the penton base gene contain hotspots for recombination in species HAdV-D [20]. Analysis of the primary amino acid sequences in species HAdV-D showed that the most similar loop1 sequences (nucleotides 200–600) to that of HAdV-D59 were HAdV-D28 and HAdV-D36 (80.95%). In addition, the most similar RGD loop (nucleotides 650–1150) to HAdV-D59 was HAdV-D22 with 100% amino acid identity. Bootscan analysis [17] with penton base sequences from species HAdV-D confirmed the aforementioned relationships (Fig. 3). Phylogenetic analysis of the HAdV-D59 penton base hypervariable loop 1 also confirmed that it is a close relative of HAdV-D28 and HAdV-D36 with a robust bootstrap value of 91 (Fig. 3B). Phylogenetic analysis also demonstrated that the HAdV-D59 RGD loop segregates with the subclade that includes HAdV-D19C and HAdV-D22 with a bootstrap value of 92 (Fig. 3C).

### Hexon Gene Analysis

**Figure 4:**
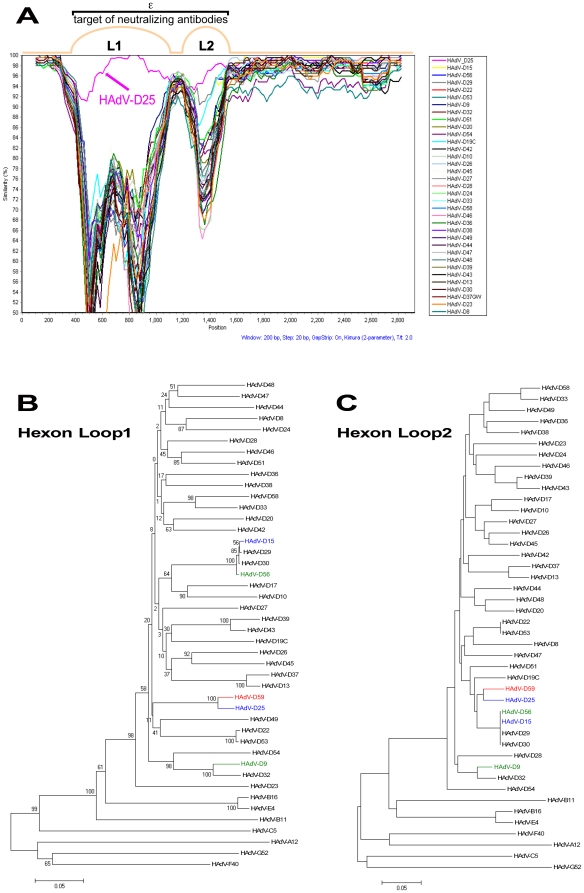
Computational analysis of the hexon gene. (A) SimPlot analysis of the HAdV-D59 hexon coding sequence. L1 and L2 correspond to loops 1 and 2 of the hexon gene, which comprise the determinant of virus serum neutralization. (B) Phylogenetic analysis of the hexon L1 sequences in species HAdV-D. (C) Phylogenetic analysis of the hexon L2 sequences in species HAdV-D. A phylogenetic tree was generated along with representatives from the other species. Phylogenetic trees were generated from aligned sequences using MEGA, via the neighbor-joining method and a bootstrap test of phylogeny.

A previous study showed that recombination in HAdVs can occur within the hexon gene of viruses in species HAdV-D [8]. This has also been demonstrated in other species of HAdVs [4], [5]. To determine whether or not the HAdV-D59 hexon coding region was either novel or the result of a recombination event, SimPlot analysis was performed. SimPlot results were not consistent with a recent recombination event in the hexon of HAdV-D59 (Fig. 4A). In addition, the nucleotide percent identity to other hexon coding sequences in species HAdV-D was determined (Table 1). HAdV-D59 loops 1 and 2 (L1 and L2) were 96.48% and 95.19% identical to L1 and L2 of HAdV-D25, respectively (Table 1). Phylogenetic analysis demonstrated that L1 of HAdV-D59 and HAdV-D25 are as distantly related as the following HAdV pairs which were shown to be distinct via serology [21]: HAdV-D39 and HAdV-D43; HAdV-D37 and HAdV-D13; HAdV-D15 and HAdV-D30; HAdV-D29 and HAdV-D30; HAdV-D9 and HAdV-D32, as well as HAdV-D45 and HAdV-D26 (Fig. 4B). Furthermore, the same phenomenon was observed for L2 with the exception of the HAdV-D45/D26 pair (Fig. 4C).

The difference in nucleotide identity between HAdV-D59 and HAdV-D25 (the nearest phylogenetic relative to HAdV-D59) in the L1 and L2 domains are greater than 2.5% (3.52% and 4.81%, respectively). Madisch et al stated that percent nucleotide identity differences greater than 2.4% and 2.5% in L1 and L2, respectively, strongly suggests identification of a novel HAdV [22]. Therefore the percent of nucleotide identity differences in L1 and L2 of HAdV-D59 further suggests that the aforementioned virus is novel.

### E3 Genome Region Analysis

**Figure 5:**
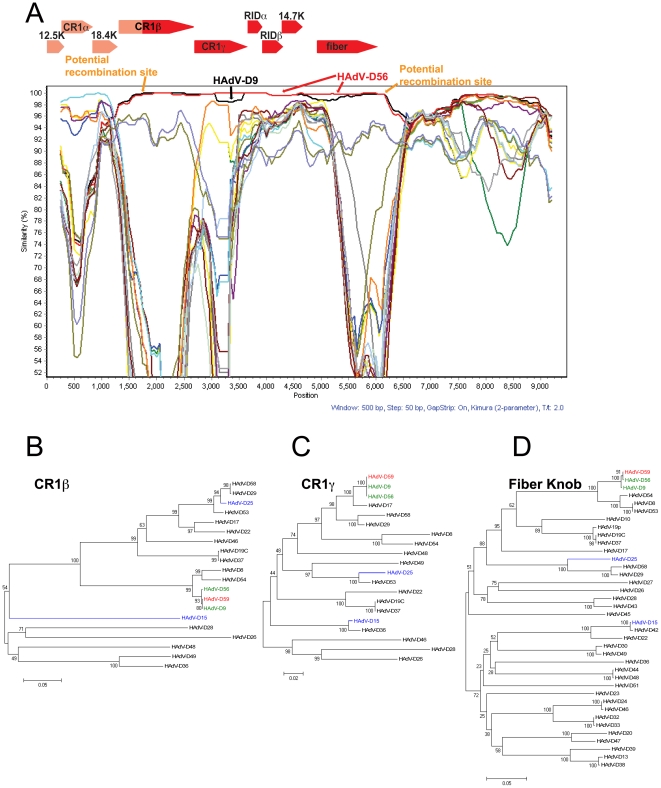
Computational analysis of the E3 and Fiber regions. (A) SimPlot analysis of the E3 and fiber region of HAdV-D59 compared to fully sequenced E3 and fiber regions from species HAdV-D. The arrows over the Bootscan demarcate the approximate positions of the E3 coding sequences. Phylogenetic analysis of (B) HAdV-D59 CR1β (C) HAdV-D59 CR1γ, and (D) fiber knob. The phylogenetic trees were generated from aligned sequences using MEGA, via the neighbor-joining method and a bootstrap test of phylogeny.

SimPlot and Bootscan results suggest that a large portion of the E3 transcription region (genes encoding for the CR1β, 18.4 k, CR1γ, RIDα, RIDβ, and 14.7 k proteins) in HAdV-D59 may have originated from a recombination event between either HAdV-D56 or HAdV-D9 and another yet to be described HAdV (Fig. 5). Interestingly, the SimPlot results suggest that a recombination event took place within the open reading frame of the CR1β gene (Fig. 5A). We also examined other E3 genes in species HAdV-D and did not detect common recombination loci (data not shown).

Phylogenetic analyses of two genes (CR1β and CR1γ genes) in the E3 region demonstrate that the coding regions for CR1β and CR1γ in HAdV-D59 are closely related to those of HAdV-D9 and HAdV-D56 (Fig. 5B, 5C).

### Fiber Gene Analysis

SimPlot analyses of the HAdV-D59 fiber was performed on fiber sequences extracted from GenBank. The results suggested that the fiber gene of HAdV-D59 is nearly identical with sequences from both HAdV-D56 and HAdV-D9 (Fig. 5A). Furthermore, the fiber of HAdV-D59 had 99.54% and 99.84% nucleotide identities with HAdV-D9 and HAdV-D56 (Table 1), respectively. Phylogenetic analysis of the fiber genes in species HAdV-D confirms that the fiber of HAdV-D59 was closest in sequence to the corresponding sequences in HAdV-D56 and HAdV-D9 (Fig. 5D).

### Serum Neutralization

HAdV-D59 was neutralized by both HAdV-D25 and HAdV-D15 antiserum, yet not by HAdV-D9 antiserum (Table 2). Interestingly, HAdV-D25 antiserum showed at least a two-fold higher neutralization titer to HAdV-D59 (greater than 1∶4096) than it did against its cognate antigen HAdV-D25 (1∶2048) (Table 2). These results demonstrate that the antigenic profile of HAdV-D59 differs from that of HAdV-D25 (Table 2). HAdV-D15 antiserum demonstrated only a two-fold increase in its ability to neutralize its cognate HAdV-D15 (1∶1024) relative to the heterologous HAdV-D25 antigen (1∶512). Similar reciprocal results were obtained with HAdV-D25 antiserum against HAdV-D15 (1∶1024) and HAdV-D25 (1∶2048). When tested with rabbit serum our results indicate that HAdV-D15 and HAdV-D25 are not as serologically distinct as previously reported [21].

**Table 2:** Neutralization of HAdV-D59 with hyper immune serum.

		Antiserum	
	αHAdV-D9	αHAdV-D15	αHAdV-D25
HAdV-D9	1∶128	<8*	<8*
HAdV-D15	<8*	1∶1024	1∶1024
HAdV-D25	<8*	1∶512	1∶2048
HAdV-D59	<8*	1∶128	>1∶4096

* No neutralization.

## Discussion

Our results demonstrated that HAdV-D25 antiserum was more effective at neutralizing HAdV-D59 than HAdV-D25 (Table 2). Since L1 and L2 protrude from the surface of HAdVs [23], it is not surprising that there is a difference between the ability of HAdV-D25 antiserum to neutralize the different viruses. One possibility for the differences in neutralization may be that the few differences in the primary amino acid structure present the HAdV-D59 hexon three-dimensional structure in such a way that the neutralizing epitopes are enhanced, thus making the virus easier to neutralize. Interestingly, we also observed a two-fold difference between the ability of HAdV-D15 and HAdV-D25 to be neutralized by reciprocal antiserum. This contradicts one study that showed antiserum to HAdV-D15 and HAdV-D25 did not cross-react in reciprocal neutralization experiments [21]. However, our data are consistent with the original characterization of HAdV-D15 and HAdV-D25 (previously called BP-1) [24], thus we conclude that HAdV-D15 and HAdV-D25 are not separate serotypes according to the traditional methods used for differentiating serotypes. These results also demonstrate that using neutralization as a criterion to type novel adenoviruses is complicated by non-standard serology methods and reagents that may yield interlaboratory variability of neutralization results. In contrast, using genomics as a method for typing HAdVs is consistent regardless of which laboratory generates the results.

**Figure 6:**
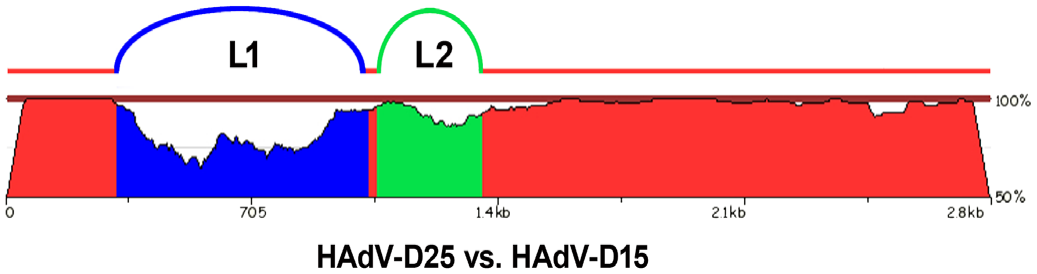
Pairwise nucleotide comparison of the hexon genes of HAdV-D15 and HAdV-D25. The blastz program zPicture was used to compare the hexon genes of HAdV-D15 and HAdV-D25. The blue region represents L1 and the green region represents L2.

Even though HAdV-D15 and HAdV-D25 showed only a two-fold difference via serum neutralization, they were recognized as different serotypes by Rosen et al, because they had different fiber proteins [24]. HAdV-D15 and HAdV-D25 share 79.13 and 92.56 percent nucleotide identity in L1 and L2, respectively. Thus, bioinformatic analysis demonstrates that they are actually different types using the criteria established by Madisch et al. which states that the nucleotide identity of L2 must differ by greater than 2.5 percent to type a novel virus [22]. Furthermore, pairwise nucleotide comparison of the hexon coding sequences for HAdV-D15 and HAdV-D25 show how genetically divergent they are (Fig. 6). Neutralization assays measure the overall effect of various antibodies that bind to multiple epitopes and may yield variable interlaboratory results due to non-standard methods and reagents. Genomic analyses measure genetic differences in the genome that have the potential to affect the pathogenicity of the virus and can be independently verified by most laboratories. The contrasting serology and genomics results for HAdV-D15 and HAdV-D25 demonstrate that these two methods do not always yield concordant results.

SimPlot analysis of the HAdV-D59 L1 and L2 regions demonstrates high nucleotide identity between the hexons of HAdV-D25 and HAdV-D59 and suggests that they may be derived from a yet undiscovered common ancestor. If L1 and L2 of HAdV-D25 and HAdV-D59 are from a common ancestor, the recombination event may be ancient, as evidenced by 3.52 and 4.81 percent nucleotide differences in the L1 and L2 sequences, respectively. If the recombination events were recent, SimPlot analysis would illustrate near 100% nucleotide identity, which was shown for HAdV-D53 and HAdV-D56 [4], [5], [19] With a distant past recombination event the hexon genes from HAdV-D25 and HAdV-D59 would have mutated over many replication cycles, after the initial recombination, to result in the variation we detected.

Multiple studies have shown that HAdVs in species HAdV-D recombine with one another in the penton base and hexon genes [4],[8],[20]. In this paper, we demonstrate that recombination may have occurred in the E3 region of HAdV-D59; however, after examining all of the sequenced E3 genes in species HAdV-D, we found that there was not a predictable pattern of recombination (data not shown). Viruses in species HAdV-D show variability in their cell tropism ranging from growth in ocular tissues to gastrointestinal and/or respiratory tissues [4], [25], [26]. Given that the fiber knob is an important determinant of cell tropism, it may be concluded that recombination is an important molecular evolution pathway for the diversity observed within species HAdV-D.

The section of the HAdV-D59, -D56, and –D9 genomes that encodes for CR1β, CR1γ, RIDα, RIDβ, 14.7K, and fiber show high nucleotide identity (Fig. 5A). From the nucleotide data, it is impossible to tell whether or not the 3′ end of HAdV-D59 came from HAdV-D56 or HAdV-D9. Although HAdV-D9 was discovered in 1957 [25], there has been no disease associated with this virus. In contrast, prior serological evidence suggests that HAdV-D56, an ocular and respiratory pathogen (with hexon and fiber coding sequences similar to HAdV-D15 and HAdV-D9, respectively) [12], [26] has been implicated in human disease as early as 1960 [27] and at other points in time as well [27], [28], [29], [30], [31], [32]. HAdV-D59 may have also existed prior to our current description yet had gone undetected during the same time periods. Thus, it is impossible to say with absolute certainty which of the aforementioned viruses existed first and/or whether they evolved from a common ancestor. Future genomic analysis of known and unknown adenoviruses is needed to elucidate further the evolutionary history of HAdVs.
